# RETRACTED: Sun et al. Resveratrol Inhibits the Migration and Metastasis of MDA-MB-231 Human Breast Cancer by Reversing TGF-β1-Induced Epithelial-Mesenchymal Transition. *Molecules* 2019, *24*, 1131

**DOI:** 10.3390/molecules30122481

**Published:** 2025-06-06

**Authors:** Yang Sun, Qian-Mei Zhou, Yi-Yu Lu, Hui Zhang, Qi-Long Chen, Ming Zhao, Shi-Bing Su

**Affiliations:** 1 Research Center for Traditional Chinese Medicine Complexity System, Shanghai University of Traditional Chinese Medicine, Shanghai 201203, China; 2 AntiCancer Inc., San Diego, CA 92100, USA

The *Molecules* journal retracts the article titled “Resveratrol Inhibits the Migration and Metastasis of MDA-MB-231 Human Breast Cancer by Reversing TGF-β1-Induced Epithelial-Mesenchymal Transition” [1], cited above.

Following the publication, concerns were brought to the attention of the Editorial Office regarding image duplications and indications of inappropriate modifications contained within this publication [1].

Adhering to our standard procedure, the Editorial Office and Editorial Board conducted an investigation that confirmed a range of image anomalies, partial duplications and indications of inappropriate modifications contained within Figures 2, 3B and 4A,C.

Despite numerous attempts to reach out to the authors and their institution for clarification on these concerns, no response has been received. Consequently, the Editorial Board has lost confidence in the reliability of the findings and decided to retract this publication [1], as per MDPI’s retraction policy (https://www.mdpi.com/ethics#_bookmark30).

This retraction was approved by the Editor-in Chief of the *Molecules* journal.

The authors did not provide a comment on this decision.

## References

[1] Sun Y., Zhou Q.-M., Lu Y.-Y., Zhang H., Chen Q.-L., Zhao M., Su S.-B. (2019). RETRACTED: Resveratrol Inhibits the Migration and Metastasis of MDA-MB-231 Human Breast Cancer by Reversing TGF-β1-Induced Epithelial-Mesenchymal Transition. Molecules.

